# Supplementary material for: Scorpion-Venom-Derived Antimicrobial Peptide Css54 Exerts Potent Antimicrobial Activity by Disrupting Bacterial Membrane of Zoonotic Bacteria
Source: Antibiotics (Basel). 2020 Nov 20;9(11):831. doi: 10.3390/antibiotics9110831 (PMC7699533; doi:10.3390/antibiotics9110831)
Supplement: Supplementary file 1 [file antibiotics-09-00831-s001.pdf]

antibiotics

MDPI

Article

Scorpion Venom-derived Antimicrobial Peptide Css 54 Exerts Potent Antimicrobial Activity by Disrupting Bacterial Membrane of Zoonotic Bacteria

Jonggwan Park[1], Jun Hee Oh [2], Hee Kyoung Kang[2], Moon-Chang Choi[2], Chang Ho Seo[1] and Yoonkyung Park[2,*]

1. Department of Bioinformatics, Kongju National University, Kongju 38065, Korea; for_quality@naver.com (J.P.); chseo@kongju.ac.kr (C.H.S.)
2. Department of Biomedical Science, Chosun University, Gwangju 61452, Korea; toqkfqkekr2@naver.com (J.H.O.), mgenetics@daum.net (H.K.K.), choist777@gmail.com (M.-C.C.)

* Correspondence: y_k_park@chosun.ac.kr; Tel.: +82-62-230-6854; Fax: +82-62-225-6758

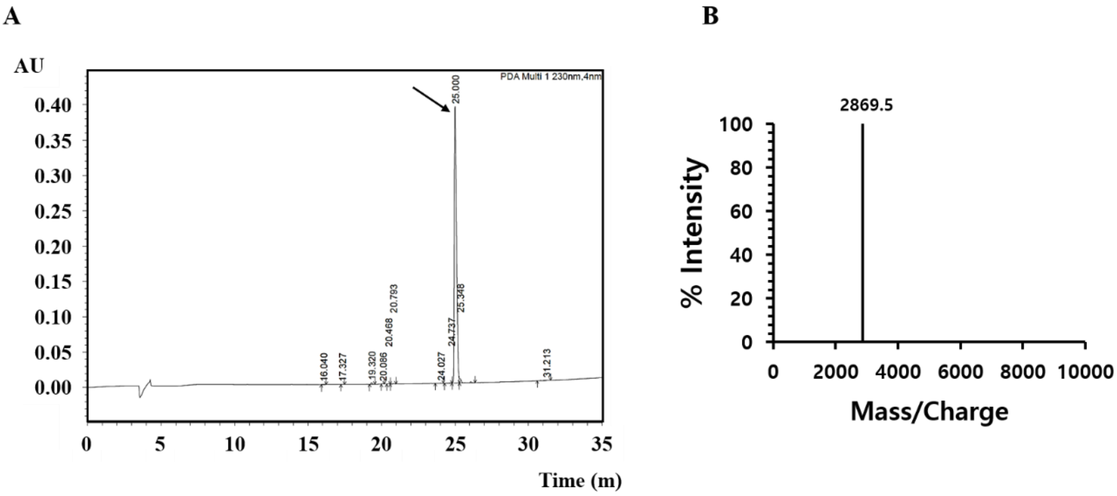

Figure S1. RP-HPLC and mass spectrometry. (A) RP-HPLC profile on a C18 column with detection at 230 nm. The black arrow represents the retention time of Css54 (25 min). (B) MALDI mass spectrometric analysis of Css54. The respective mass/charge ratio was 2869.5.

© 2020 by the authors. Submitted for possible open access publication under the terms and conditions of the Creative Commons Attribution (CC BY) license (http://creativecommons.org/licenses/by/4.0/).
